# Decellularization techniques: unveiling the blueprint for tracheal tissue engineering

**DOI:** 10.3389/fbioe.2025.1518905

**Published:** 2025-02-28

**Authors:** Keisha T. Gomes, Palla Ranga Prasad, Jagnoor Singh Sandhu, Ashwini Kumar, Naveena A. N. Kumar, N. B. Shridhar, Bharti Bisht, Manash K. Paul

**Affiliations:** ^1^ Department of Radiation Biology and Toxicology, Manipal School of Life Sciences, Manipal Academy of Higher Education, Manipal, India; ^2^ Central Animal Research Facility, Kasturba Medical College, Manipal Academy of Higher Education, Manipal, India; ^3^ Center for Animal Research, Ethics and Training (CARET), Manipal Academy of Higher Education, Manipal, Karnataka, India; ^4^ Department of Forensic Medicine, Kasturba Medical College, Manipal Academy of Higher Education, Manipal, Karnataka, India; ^5^ Department of Surgical Oncology, Kasturba Medical College, Manipal Academy of Higher Education, Manipal, Karnataka, India; ^6^ Department of Pharmacology and Toxicology, Obscure Disease Research Center, Veterinary College Campus, Shivamogga, Karnataka, India; ^7^ Department of Microbiology, Kasturba Medical College, Manipal Academy of Higher Education, Manipal, Karnataka, India; ^8^ Division of Pulmonary and Critical Care Medicine, David Geffen School of Medicine, University of California Los Angeles (UCLA), Los Angeles, CA, United States

**Keywords:** trachea, decellularization, recellularization, scaffolds, transplantation

## Abstract

Certain congenital or acquired diseases and defects such as tracheo-oesophageal fistula, tracheomalacia, tracheal stenosis, airway ischemia, infections, and tumours can cause damage to the trachea. Treatments available do not offer any permanent solutions. Moreover, long-segment defects in the trachea have no available surgical treatments. Tissue engineering has gained popularity in current regenerative medicine as a promising approach to bridge this gap. Among the various tissue engineering techniques, decellularization is a widely used approach that removes the cellular and nuclear contents from the tissue while preserving the native extracellular matrix components. The decellularized scaffolds exhibit significantly lower immunogenicity and retain the essential biomechanical and proangiogenic properties of native tissue, creating a foundation for trachea regeneration. The present review provides an overview of trachea decellularization advancements, exploring how recellularization approaches can be optimized by using various stem cells and tissue-specific cells to restore the scaffold’s structure and function. We examine critical factors such as mechanical properties, revascularization, and immunogenicity involved in the transplantation of tissue-engineered grafts.

## 1 Introduction

The trachea is a crucial part of the respiratory system, forming a path that allows airflow into the lungs, facilitating gaseous exchange and blood oxygenation. Structurally, it is a hollow, semi-flexible, tube-like structure extending about 10–13 cm. It is formed by 18–22 C-shaped cartilaginous rings connected by a thin fibroelastic membrane (Furlow and Mathisen, 2018). The tracheal architecture comprises four layers: mucosa, submucosa, musculo-cartilaginous, and adventitia. Each layer has its distinct characteristics and functions (Brand-Saberi and Schäfer, 2014). Various congenital or acquired diseases and defects such as tracheo-oesophageal fistula, tracheomalacia, tracheal stenosis, airway ischemia, and tumors can affect the physiology of the trachea, which is a major health concern (Melgarejo-Ramírez et al., 2022). For example, tracheoesophageal fistulas commonly occur in conjunction with esophageal atresia, creating an abnormal link between the trachea and esophagus, disrupting both respiratory and digestive systems (Ke et al., 2015). Tracheomalacia weakens the walls of the trachea, leading to airway collapse during breathing, while tracheal stenosis can result from prolonged intubation or inflammation, which narrows the airway and restricts airflow, potentially causing upper airway obstruction (Wain, 2009; Poornejad et al., 2016). Additionally, tracheal tumors, whether benign or malignant, can obstruct airflow and invade surrounding tissues (Grillo and Mathisen, 1990). Different surgical procedures are used to treat tracheal disorders. However, these procedures only offer temporary relief, do not fully restore function, and can lead to other complications. Additionally, no surgical treatments are available for long-segment defects that affect at least half of an adult’s total tracheal length or at least a third of a child’s total tracheal length (Jiwangga et al., 2024a). Thus, researchers began exploring alternative approaches to overcome these hurdles.

Tissue engineering (TE) is a multidisciplinary field that aims to address the limitations of traditional surgical methods by developing biocompatible grafts. These 3D biomimetic and functional tissue-like substitutes not only repair but also regenerate damaged/injured and diseased tracheal tissue. The three fundamental pillars of tissue engineering include scaffolds, cells, and signaling molecules (Gilpin and Yang, 2017). Stem cells like induced pluripotent stem cells (iPSCs), mesenchymal stem cells (MSCs), and embryonic stem cells (ESCs) have the potential to differentiate into various cell types found in the trachea, making them suitable for tracheal tissue engineering (Olson et al., 2011). The use of appropriate scaffolds is crucial as they offer mechanical support, provide biochemical cues, and have an impact on tracheal regeneration. These scaffolds are designed to mimic the structure and properties of the extracellular matrix (ECM) of native tissues. Additionally, the selection of signaling molecules is crucial for tracheal regeneration, including cytokines, growth factors, and other bioactive ingredients that are required to control cell behaviour and tissue formation.

Recent advances in tissue engineering methods like 3D bioprinting and electrospinning allow for the precise fabrication of synthetic scaffolds using various polymers and hydrogels. Despite the advances in this field, mimicking the complex microenvironment and regional structural variations of the trachea remains challenging, as tracheal tissues have a very complex ECM composed of collagens, elastin, and elastic fibers, proteoglycans (PGs) and glycosaminoglycans (GAGs), laminins, fibronectin, and other proteins and glycoproteins, along with an even more intricate vascular arrangement (Karamanos et al., 2021). Using a natural scaffold harvested from either human or animal sources would help overcome some of these issues. Thus, current research is oriented towards the use of the natural ECM to produce biological tracheal scaffolds. This natural ECM can be achieved through decellularization. It is an important technique that removes the cellular and nuclear components from tissue while preserving the native ECM (Choudhury et al., 2020). It eliminates the major histocompatibility complexes such as MHC-I and MHC-II that activate the immune response-inducing systems of the trachea while preserving the ECM’s microarchitecture (Batioglu-Karaaltin et al., 2019). Decellularization can be achieved through various physical, chemical, and enzymatic methods often used in combination. Detergent-enzymatic method (DEM), laser micropore technique, and vacuum-assisted decellularization (VAD) are some commonly used approaches. The resulting acellular scaffolds exhibit minimal immunogenicity and are easily identified by the body. They retain the essential biomechanical and proangiogenic properties of native tissue, creating a foundation for tracheal regeneration (Neishabouri et al., 2022). Moreover, these scaffolds have shown promising biocompatibility and support for cell growth.

In addition to decellularization, scaffold recellularization is also crucial for tracheal regeneration, involving the introduction of cells into the scaffold, commonly employs stem cells. The cells can be allogeneic (from a donor), autologous (from the patient), or xenogeneic (from another species) sources. In the native trachea, chondrocytes form the hyaline tracheal cartilage, while the inner surface is lined with mucociliary epithelium. Mesenchymal stem cells (MSCs) derived from adipose tissue (AT-MSCs) or bone marrow (BM-MSCs) are frequently used to generate chondrocytes. While, protocols exists for the generation of iPSC-derived or airway basal stem cell (ABSC)-derived airway epithelial cells, which can aid in epithelial regeneration (Batioglu-Karaaltin et al., 2019). Various cell-seeding techniques are employed, including direct injection of chondrocytes into tracheal rings, laser perforation to enhance cartilage porosity, fibrin encapsulation for uniform cell attachment, and methods such as static seeding, hydrogel encapsulation, and perfusion recellularization (Milian et al., 2021; de Wit et al., 2023). Achieving sufficient revascularization remains challenging but is essential to prevent necrosis, infection, and graft failure. Optimized use of cytokines, growth factors, and bioactive molecules can facilitate vascular network reconstruction (Neishabouri et al., 2022). Recent advancements in TE are significantly improving the possibility of tracheal transplants by addressing critical challenges such as successful recellularization, revascularization, mechanical properties, and epithelialization. Various decellularization techniques have been developed, but none of them have demonstrated complete success in the removal of cells and the retention of the ECM and mechanical properties. This review highlights advancements in trachea decellularization and explores optimized recellularization strategies using stem and tissue-specific cells to restore scaffold structure and function, focusing on mechanical properties, revascularization, and immunogenicity critical to successful tissue-engineered graft transplantation.

## 2 Tracheal morphology

The distal respiratory system consists of the trachea and the bronchi (Jain and Sznajder, 2007). The trachea is a complex organ and plays a vital role in the physiological function of respiration, facilitating gaseous exchange and ultimately blood oxygenation via the lungs. The tracheal epithelium acts as a barrier, protecting the airways from respiratory microbes. The mucus in the airway lubricates the surface and captures infectious particles, pathogens, and other airborne agents during inflammation (Mammana et al., 2024). Their functions are closely linked to their structures. Structurally, the trachea is a hollow, semi-flexible tube-like structure and extends to around 10–13 cm in length. The trachea begins at the cricoid cartilage’s lower end and terminates at the carina (Furlow and Mathisen, 2018). Females usually have a shorter trachea. It extends from the sixth cervical vertebra to the fourth thoracic vertebra and then bifurcates into two main bronchi. Anatomically, the trachea has a characteristic D-shaped cross-section and is formed by 18–22 cartilage rings, connected by a thin fibroelastic membrane. The C-shaped cartilaginous rings are assembled one above the other on the anterior side, joint by a membranous wall with the trachealis muscle on the posterior side. The trachealis muscle is located adjacent to the esophagus. This muscle serves as the active component of the trachea, demonstrating responsiveness to external stimuli. It helps in tracheal contraction to allow coughing and swallowing (Teng et al., 2012). Each ring has a height and thickness of about 4 mm and 3 mm, respectively, with an outer diameter of approximately 2–2.2 cm in men and 1.8–2 cm in women. The cartilage rings maintain the trachea’s structure despite fluctuations in intrathoracic pressure during respiration. The inter-cartilaginous membrane connects the upper and lower rings. The tracheal architecture comprises four layers: mucosa, submucosa, musculo-cartilaginous, and adventitia (Jiwangga et al., 2024a). The mucosal layer surrounds the lumen and has ciliated, epithelial cells, basal stem cells, goblet cells, serous cells, club cells, and neuroendocrine cells, all supported by the lamina propria, which houses the tracheal or submucosal glands (SMG). The mucociliary escalator performs innate defence function by removing bacteria, fungus, pathogens, allergens, and other particles. The submucosa majorly consists of globular cells, nervous tissue, mucous glands, and blood vessels. The musculo-cartilaginous layer consists of tracheal muscle, fibroelastic tissue, and cartilaginous plates of hyaline cartilage surrounded by perichondrium, which plays a critical role in facilitating chondrocyte growth (Yoon et al., 2013). Collagen, present in the cartilage, is a major biomechanical component that makes the trachea rigid laterally, but flexible longitudinally. The elastic fibers are well organized, unlike the collagen fibers, which are not oriented. The adventitial layer is the outermost and made up of connective tissues.

The tracheal ECM, produced by native cells, plays a vital role in supporting cell growth, maintaining homeostasis, and ensuring the structural and functional integrity of the airway (Ravindra et al., 2021). It is a highly specialized structure composed of collagen, elastin, glycosaminoglycans (GAGs), glycoproteins, and bioactive molecules, each contributing to the trachea’s mechanical, structural, and biochemical properties (Yoon et al., 2013; Mason and Wusteman, 1970). Collagen, the most abundant ECM component, provides tensile strength and flexibility through type I in the submucosa and adventitia, type II, III, in cartilage integrity, type III in submucosal and lamina propria support, and type IV in the basement membrane for epithelial anchoring (Liu et al., 2021a). Elastin offers essential elasticity and flexibility, enabling the trachea to stretch and recoil during breathing while maintaining airway integrity (Kamel et al., 2009). GAGs such as hyaluronic acid and chondroitin sulfate retain hydration, enhance compressive strength, and support cellular signaling (Deņisova et al., 2022). Glycoproteins, including fibronectin and laminin, facilitate cellular adhesion, repair, and polarity maintenance (Deņisova et al., 2022). Bioactive molecules like cytokines, growth factors, and matrix metalloproteinases regulate cell proliferation, immune responses, and ECM remodeling, ensuring efficient tissue repair and resilience during respiration (Feng et al., 2023). Tracheal vascularisation occurs in a segmental fashion. The segmental arteries branch into anterior and posterior transverse inter-cartilaginous arteries, that are distributed circumferentially. Based on the vascularisation, the trachea is split into the upper cervical and lower thoracic regions. The first tracheoesophageal branch supplies blood to the lower cervical trachea. While the second and third branches deliver blood to the middle and upper cervical trachea, respectively. The bronchial arteries supply blood to the thoracic trachea.

Tracheal tumors, tracheo-oesophageal fistula, tracheomalacia, and tracheal stenosis are common tracheal disorders needing surgical intervention (Macchiarini et al., 2006; Wert, 2004; Judd et al., 2010; Wain, 2009). Diseases that affect the major airways pose a danger to life expectancy and quality of life. Respiratory diseases and disorders are one of the main causes of death in the US and around the world today (Balestrini et al., 2015). Such damage may result from inflammatory reactions, infections, congenital defects, external trauma, and injury during surgical procedures. Smoking is the primary cause of tracheal tumors (Harris et al., 2018). Various surgical procedures are employed to treat tracheal disorders. These include techniques such as bronchoscopic dilation, tracheobronchial airway stent, tracheostomy, tracheoplasty, tracheal resection, and reconstruction (McNamara and Crabbe, 2004; Perez Holguin and Reed, 2023). These surgical procedures do not permanently restore function and can lead to further complications. Moreover, long segment defects in at least half the total length of tracheas in adults and at least a third of the total trachea’s length in children have no available surgical therapies and treatments (Jiwangga et al., 2024a). Despite significant efforts, achieving a fully functional trachea is complex and challenging, (Kotloff and Thabut, 2011). The growing need for transplantable organs in end-stage organ failures has prompted researchers to look into novel solutions. The airway tissues are fragile and frequently compromised during the transplantation and procurement processes. Common complications include primary graft dysfunction, airway ischemia, bronchial stenosis, infections, and acute rejections (Kotloff and Thabut, 2011). This is largely due to the complexity of the ECM and the intricate gross anatomical structure of the trachea, both of which are essential for maintaining tracheal function. The components of the ECM provide mechanical strength, elasticity, and biochemical signals vital for cellular behavior and tissue repair (Lei et al., 2021). Meanwhile, the anatomical features of the trachea such as vascularized mucosa, the posterior membranous wall, and C-shaped cartilage rings ensure flexibility, facilitate dynamic respiratory movements, and help to maintain airway patency (Minnich and Mathisen, 2007). Replicating these features in engineered constructs requires preserving the biomechanical properties of cartilage, the flexibility of the membranous wall, and the regenerative potential of the mucosa (Rhodin, 1966). Additionally, the ECM’s zonal heterogeneity is also crucial for cell attachment and integration with host tissues (Jiwangga et al., 2024a). Therefore, developing a scaffold that mimics these characteristics while ensuring proper vascularization, immune compatibility, and sustained functionality remains a significant challenge in creating effective tracheal treatment solutions (Wei et al., 2024). The advancement of bioengineered organs presents a promising potential solution. Preserving the key components such as zonal ECM heterogeneity, epithelial basement membrane, vascularized mucosa, cartilage rings, posterior membranous wall, and tracheal geometry is crucial for maintaining site-specific mechanical properties, epithelial repair, nutrient delivery, airway patency, flexibility, and mucociliary clearance (Hong et al., 2018). By maintaining these characteristics, engineered tracheal substitutes can effectively mimic the native trachea, restoring proper airway functionality. Thus, the development of bioengineered organs would provide a potential solution. Tissue engineering has gained popularity in regenerative medicine, offering technology to create biological constructs that can mimic and enhance the functions of the trachea.

## 3 Decellularization

Nowadays, tissue engineering techniques show promise in creating artificial tracheal grafts with organ-specific functions (Park et al., 2015; Taniguchi et al., 2018). The three main components of tissue engineering are cells, scaffolds, and growth factors, which are necessary to create the desired tissue (Machino et al., 2019). In addition, various fabrication methods have been used in tracheal tissue engineering, including decellularization, 3D bioprinting, and electrospinning, to repair the injured/damaged tracheal tissues. In tissue engineering, the scaffold is an important component for the success of the biological construct. The vast diversity in the tracheal cell types poses a challenge to developing a scaffold capable of supporting the differentiation and growth of all the cell types. The tissue-engineered graft should match the organization and orientation of all the elastin and collagen fibers to be able to mimic the structural properties of the native trachea. Various materials should be considered while designing the graft to match the anatomical structure of each layer. Moreover, the complexity of seeding cells into the deeper layers, such as submucosa and cartilage, should also be considered while designing the scaffold (Tchoukalova et al, 2018).

Various types of scaffolds such as natural, synthetic, and hybrid scaffolds can be used for tracheal tissue regeneration (Machino et al., 2019). Among these approaches, decellularization of the trachea would be the best approach to produce a scaffold having ideal biomechanical, biocompatible, and proangiogenic properties similar to those of native tissues. Decellularization aims to remove the immune-stimulating elements of the scaffold while preserving the extracellular matrix components, which are primarily responsible for cell growth, maintenance, and regeneration. It eliminates the major histocompatibility complexes I and II (MHC-I and MHC-II) that activate the immune response-inducing systems of the trachea (Batioglu-Karaaltin et al., 2019). Thus, decellularization preserves the architecture and composition of the extracellular matrix (ECM), and the mechanical and bioactive properties of the tissues. Preservation of certain architectural components is necessary to generate a viable scaffold. The mechanical function of cartilage is defined by the properties of the matrix rather than those of the cells (Trabelsi et al., 2010). Thus, it should not get damaged during decellularization. Additionally, the annular ligaments facilitate longitudinal extension and bending and should also be kept intact to allow for neck movements, diaphragm contraction, and lung inflation (Boazak and Auguste, 2018). Other essential structures is the basement membrane to support epithelialization (Faulk et al., 2014). The following criteria are put forward by Crapo and colleagues to assess the decellularization: DNA quantification of the tissue must show ≤50 ng/mg, the DNA fragment should be lower than 200 bp and there should be a lack of nuclei visible in tissue sections stained with H&E or 4’,6-diamidino-2-phenylindole (DAPI) (Crapo et al., 2011). These properties can be achieved by using different methods.

### 3.1 Decellularization agents

The choice of decellularizing agents is vital, as it directly impacts the quality and functionality of the native ECM (Poornejad et al., 2016). The methods are chosen based on the tissue and proposed application. These can be broadly categorized into three groups: chemical, physical, and biological methods.

#### 3.1.1 Chemical methods

These include the use of acids, bases, organic diluents, detergents, chelators, toxins, hypertonic and hypotonic solutions (Crapo et al., 2011; Poornejad et al., 2016). Peracetic acid and hydrochloric acid (HCl) can be used to disrupt cell membranes and nucleic acids. Poornejad et al. (2016) found that peracetic acid is an effective way to preserve collagen fibers and tissue structure. It is mostly used in sterilization (Reing et al., 2010). Bases (calcium hydroxide and sodium hydroxide) are useful in removing immunogens but can break the collagen crosslinks and eliminate the growth factors from the ECM (Sengyoku et al., 2018). Ionic (Sodium Dodecyl Sulfate (SDS) and Sodium deoxycholate (SDC)), zwitterionic ((3-[(3-cholamidopropyl) dimethylammonio]-1-propanesulfonate) (CHAPS)), and non-ionic detergents (Triton-X100) are the most commonly used chemical agents (Jiwangga et al., 2024a). While Triton X-100 is capable of efficiently eliminating cell remnants from thicker tissues, CHAPS is preferred for the decellularization of thinner tissues (Crapo et al., 2011). Non-ionic detergents possess no ionic charge and hence have the least effect on the structure of proteins. SDS treatments are usually used in decellularization because of their ability to remove cellular material efficiently (Mendibil et al., 2020). Supercritical fluids are currently being investigated as an alternative to detergent-based decellularization (de Wit et al., 2023). They have low viscosity and are made up of inert material such as carbon dioxide. It is used for cell removal with minimal modification to the mechanical properties of the ECM. Additionally, after decellularization, tissues can be obtained in a dry state, negating the requirement for lyophilization (Topuz et al., 2020). Latrunculin B is another alternative to detergent methods. This naturally occurring toxic substance, obtained from marine sponges, acts as a potent actin cytoskeleton disruptor. Results showed that no intact nuclei were seen in the skeletal muscle after latrunculin B treatment (Reyna et al., 2020). A sequential treatment of hypertonic and hypotonic solutions is used to induce osmotic shock for the removal of cellular debris from the tissues (Milian et al., 2021). Chelators such as EDTA (ethylenediaminetetraacetic acid) release the cells from the ECM by removing certain divalent cations that facilitate the cell-ECM adhesion (Kasturi and Vasanthan, 2024). Organic diluents such as alcohol, 1% tributyl phosphate, and acetone can disrupt the cell membrane and are used for the decellularization of solid tissues. Ethanol and methanol are also used to wash off any leftover nucleic acids in the tissue (Kim et al., 2022). A major issue is its potential to fix tissues and precipitate proteins, so alcohol should be used sparingly in treating tissues.

#### 3.1.2 Physical methods

These methods comprise perfusion, lyophilization, freeze-thawing, mechanical pressure, electroporation, sonication, vacuum assistance, immersion, and agitation. Since these methods use temperature and pressure instead of chemicals, they are preferred as there is no risk of toxicity (Rabbani et al., 2021). Using a pump, perfusion creates a simulated flow of chemical and biological substances throughout the whole vasculature of the organ. It is useful for decellularization of large organs. However, it cannot digest tissues if the intrinsic vasculature is absent (Milian et al., 2021). The freeze-thaw technique uses cycles of alternating freezing and biological temperature to induce intracellular crystals. This results in cell lysis but does not eliminate the immunogenic cell remnants. The decellularization process can be enhanced by repeated freeze-thaw cycles, but this also weakens and damages the ECM (Neishabouri et al., 2022). A more common decellularization method involves immersing the tissues in solutions made with decellularization agents while undergoing constant mechanical agitation. This technique is useful for small and thin tissues, without intricate vasculature. It causes cells to rupture and detach from the basement membrane. Agitation also ensures a more homogenous exposure to the chemical detergents and reduces the decellularization time. The duration and intensity of agitation are determined by the tissue’s thickness and the detergent’s strength (Rabbani et al., 2021). Sonication induces shear stress within the cell membranes by producing vibrations. It also enhances the penetration of the detergents. However, this technique can also damage the ECM’s structural fibers (Zhang et al., 2022). Butler et al. (2017) compared an established detergent-enzymatic method (DEM) to a novel vacuum-assisted decellularization (VAD) method. They performed the decellularization of the human trachea in a vacuum environment of 1 Torr. The VAD technique showed accelerated results and lower DNA content.

#### 3.1.3 Enzymatic methods

Enzymatic agents are preferred for their high specificity. Proteases including trypsin and dispase are used for the decellularization process. Trypsin is an effective decellularization agent commonly used alongside EDTA but has the potential to damage collagen (Rahman et al., 2018). Dispase II is used to selectively cleave fibronectin and collagen IV found in the basement membranes. However, prolonged exposure can remove these elements from the ECM as well (Crapo et al., 2011). Phospholipase A2 is an esterase commonly used in decellularization. It preserves the collagen while damaging the phospholipid components (Neishabouri et al., 2022). Exo and endonucleases are often used in later steps for the removal of DNA and RNA from the tissue (Yi et al., 2017). These include DNase, RNase and Benzonase.

## 4 Advances in tracheal decellularization

**FIGURE 1:**
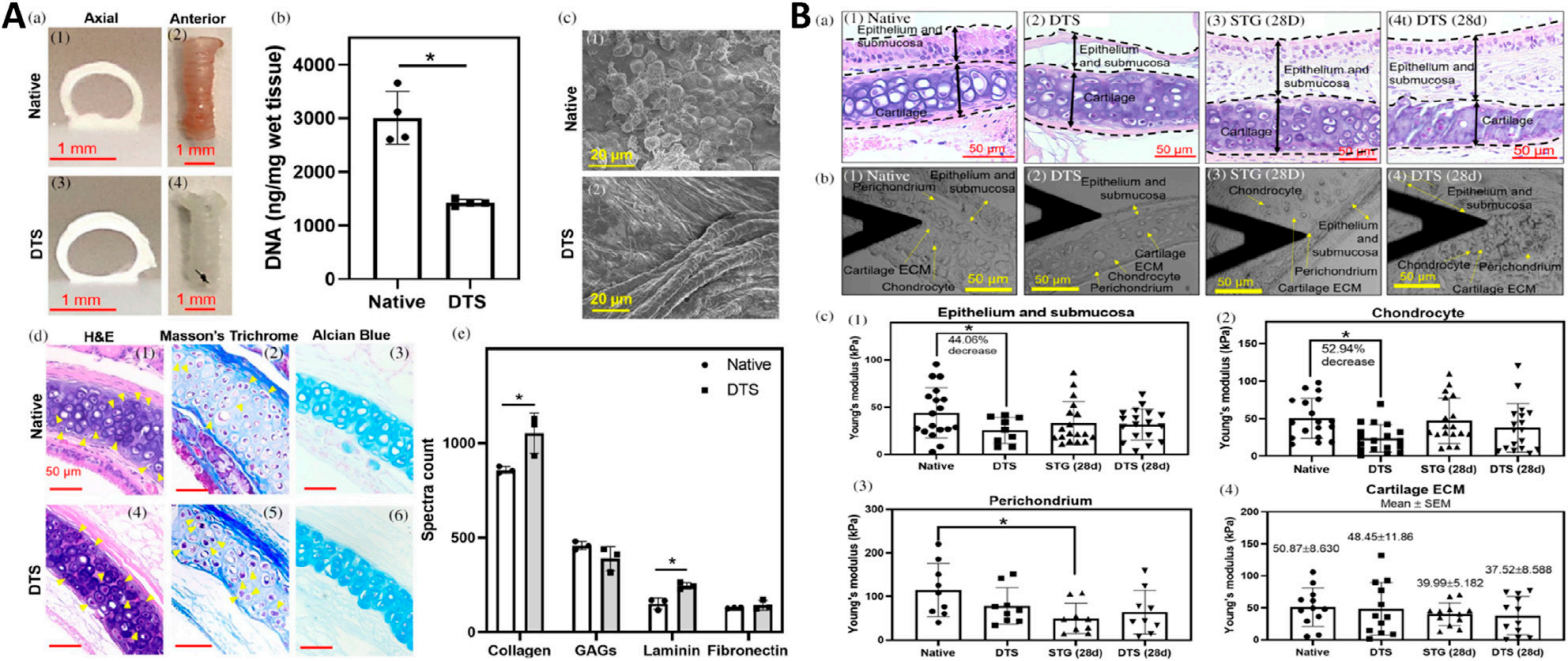
Characterization of partially decellularized tracheal scaffolds (DTS) and their regenerative potential post-orthotopic tracheal transplantation in a mouse model. **(A)** a) Photographs show axial and anterior views of native trachea and DTS. b) DNA quantification of native and DTS in wet tissue (ng/mg). c) Scanning Electron Microscopy (SEM) images of native trachea and DTS (scale bar – 20 μm). d) Histological analysis through H&E, Alcian blue, and Masson’s Trichrome staining of native trachea and DTS (yellow solid arrows indicate chondrocytes) (scale bar – 50 μm). e) Mass spectrometry spectral count for collagen, glycosaminoglycans (GAGs), laminin, and fibronectin in the native trachea and DTS. **(B)** a) H&E images of the native trachea, DTS, STG (28d), and DTS (28d), showing boundary regions (scale bar – 50 μm). b) Atomic Force Microscopy (AFM) images of tissue sections from native trachea, DTS, STG (28d), and DTS (28d) (scale bar – 50 μm). c) Measurement of Young’s modulus for epithelium and submucosa, chondrocyte, perichondrium, and cartilage ECM across native trachea, DTS, STG (28d), and DTS (28d) (Liu et al., 2021b).

An ideal decellularized tracheal scaffold should mimic the structure and functionality of the native trachea. It should have pore sizes between 5 and 100 μm, which allow for improved vascularization and cell survival (Roberts et al., 1997). To maintain optimal functioning and prevent collapse, an appropriate scaffold should also have sufficient mechanical strength (212 ± 18 N) and flexibility (10.6 ± 1.8 MPa). It should also be biodegradable, biocompatible, non-toxic, and non-immunogenic (Roberts et al., 1997). A decellularization is considered effective when the dry tissue content is less than 50 ng/mg (Crapo et al., 2011). These properties can be achieved by using different methods. In 1998, Tojo et al. (1998) performed tracheal allotransplantation in dogs without using immunosuppressive agents. They compared cryopreserved and fresh allografts. All dogs with cryopreserved allografts survived, while those with fresh allografts suffered from airway obstruction. This showed that the cryopreservation technique can reduce the immune response and antigenicity. In 2000, Liu et al. (2000) confirmed that the tracheal epithelium is a key link in causing allograft rejection (Liu et al., 2000). They harvested tracheas from dogs and treated them with 3% saline and 1% Triton X-100. Their study proved that removing the tracheal epithelium and glands can lower the antigenicity of the trachea. Cryopreserved tracheal allografts have been discovered for their reduced antigenicity, but the growth of these grafts has not yet been investigated (Figure 1). In 2003, Tanaka et al. (2003) transplanted tracheal grafts into growing rabbits (3–9 months old) and observed no significant growth of the tracheal allografts. In 2005, Conconi et al. (2005) obtained porcine tracheal scaffolds via a detergent enzymatic method using 4% sodium deoxycholate (SDC) treatment for 4 h followed by DNase I.

These tracheal matrices could support the adhesion of epithelial cells and chondrocytes and reduce MHC antigen expression. In 2009, Jungebluth et al. (2009) harvested tracheal segments from Yorkshire boars which were then subjected to the detergent–enzymatic method discussed above. These were allografted in pigs and xenografted in mice. The bioengineered tracheas displayed an absence of anti-pig leukocyte antigen antibodies and showed no upsurge in IgG and IgM content in mice. In 2010, Baiguera et al. (2010) used a detergent-enzymatic method to attain decellularization of the human trachea. The tissue was subjected to sequential treatments with 4% SDC and DNase-I (2000 KU) to destroy all nuclear and cellular content. 25 cycles of treatment were done to produce a thoroughly decellularized scaffold. The findings demonstrated that some chondrocytes were still present in the cartilaginous area even though 99% of the DNA had been removed.

In 2012, Zang et al. (2012) employed a modified detergent-enzymatic method (DEM) to decellularize tracheas harvested from Brown Norway rats. The tissue was agitated in sterile distilled water for 72 h, followed by a 4 h sodium deoxycholate (SDC) treatment. It was then subjected to 1 mM sodium chloride (NaCl) solution containing 50 kU/mL of DNase I (deoxyribonuclease I) for 3 h at 37°C and distilled water for 41 h at 4°C. A uniaxial tensile test revealed that the fresh and decellularized tracheae had similar strain at failure. Moreover, there was no statistically significant drop observed in the ultimate tensile strength following decellularization. Studying the changes in decellularized tissue over time is important to understand how the bioengineered transplant will function during the individual’s lifetime. Baiguera et al. (2012) decellularized human tracheal tissues using 25 DEM cycles (detergent-enzymatic method). The decellularized tissues were cross-linked in 1% w/v aqueous genipin solution to compensate for the GAGs lost during the process. Even so, there was no significant improvement in the mechanical properties over 1 year, suggesting that ECM will eventually deteriorate irreversibly. In 2013, Partington et al. (2013), optimized the DEM to decellularize cadaveric tracheas from pigs. 25 cycles of DEM (detergent-enzymatic method) were done using SDC and DNase. The submucosal part was completely decellularized, while chondrocytes persisted in the cartilage. These chondrocytes showed apoptosis. The laminin and fibronectin remain unaffected, whereas type II collagen, soluble collagen, and GAG content were reduced during decellularization. In 2014, Baiguera et al. (2014) developed a dynamic decellularization process for rat tracheal matrices using a detergent-enzymatic method (DEM) within a bioreactor. Post-decellularization, the tracheal matrices were cross-linked with genipin to improve mechanical properties. The dynamic process achieved complete decellularization in six cycles, significantly reducing the time needed compared to traditional methods.

**FIGURE 2:**
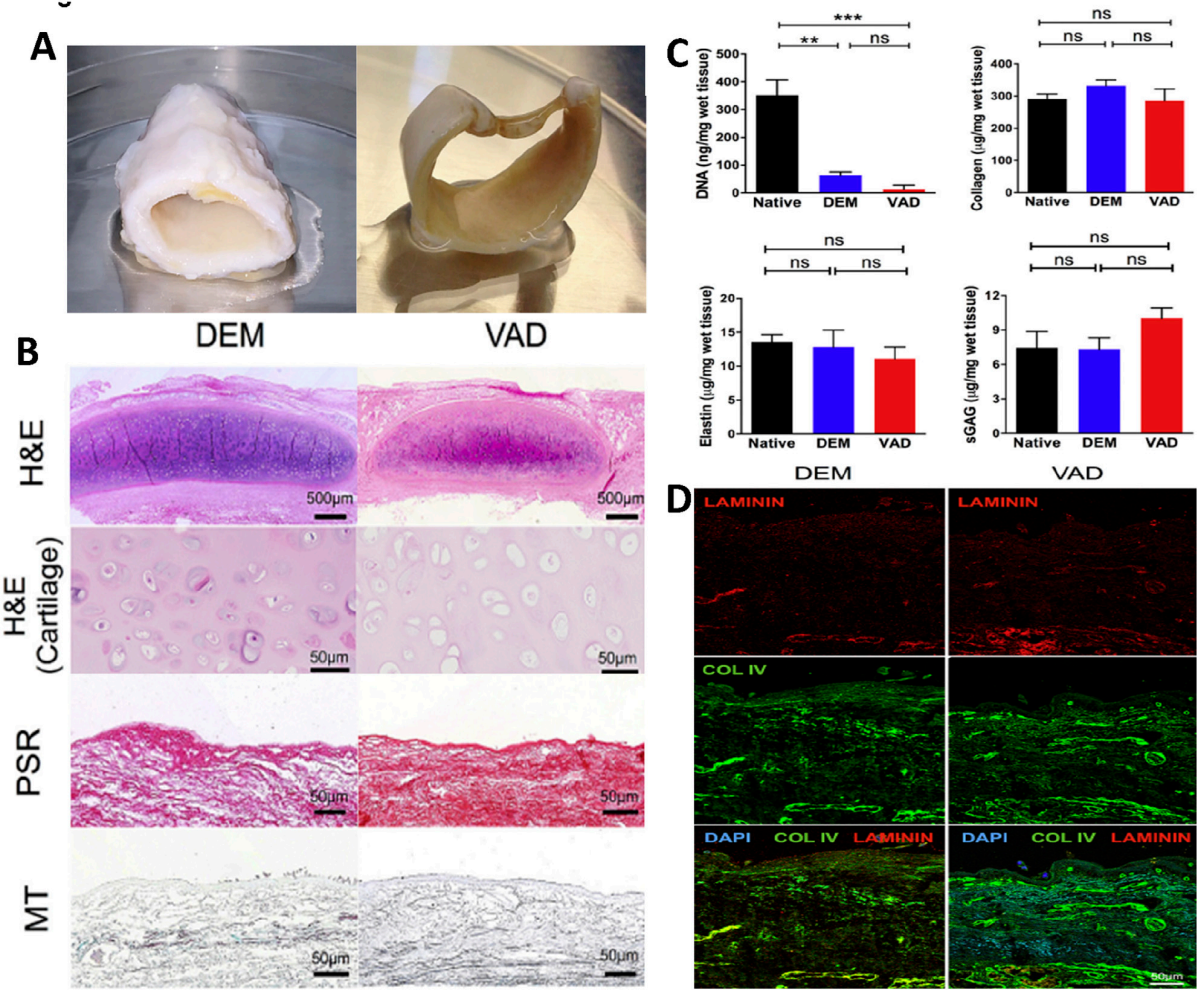
A comparative analysis of detergent-enzymatic and vacuum-assisted decellularization techniques to create tissue-engineered human tracheal scaffolds. **(A)** A gross picture of the decellularized human tracheal scaffold using detergent-enzymatic (DEM) and vacuum-assisted (VAD) techniques. **(B)** Histological analysis using H&E, Picrosirius red (PSR), and Masson’s trichrome (MT) staining to assess the structural integrity of DEM and VAD decellularized tracheal scaffolds (scale bar – 500 μm and 50 μm). **(C)** Biochemical analysis including DNA quantification, collagen and elastin estimation, and sulfated glycosaminoglycans (sGAGs) quantification of DEM and VAD-prepared tracheal scaffolds compared with the native trachea, with statistical analysis performed using the Kruskal–Wallis (KW) test. **(D)** Immunofluorescence staining of DEM and VAD-based tracheal scaffolds to reveal the presence of laminin (red) and collagen IV (green), which are counterstained with DAPI (blue) (scale bar - 50 μm) (Butler et al., 2017).

**FIGURE 3:**
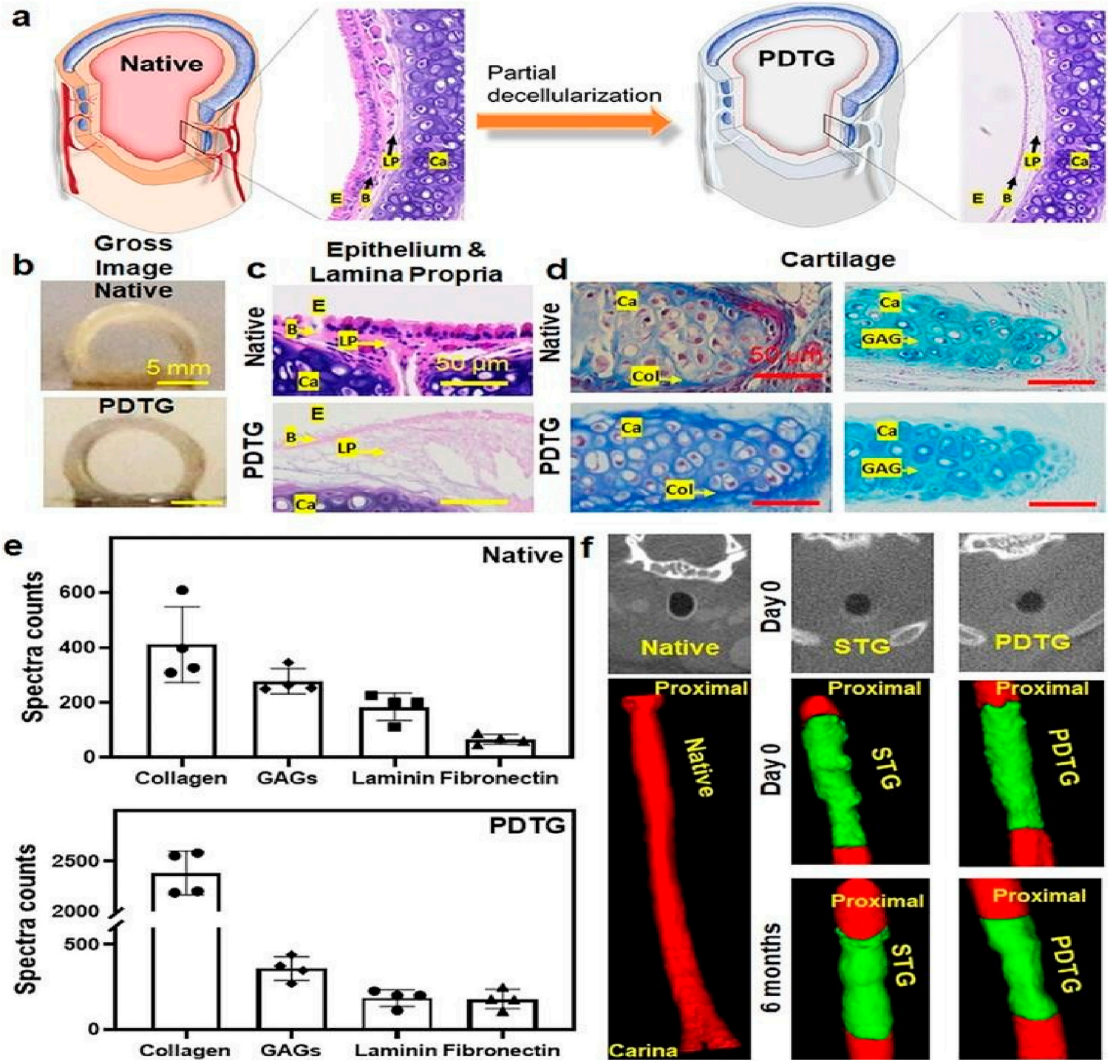
Characterization of a tracheal graft after partial decellularization. **(A)** Diagram showing the native trachea and the partially decellularized tracheal graft (PDTG) in a mice model. **(B, C)** Gross image and H and E staining of the native trachea and PDTG, indicating the removal of cells from the inner surface of the epithelium and lamina propria during the partial decellularization process. **(D)** Masson’s trichrome and Alcian blue staining of the tracheal cartilage demonstrate the ECM component’s preservation through partial decellularization. **(E)** Mass spectrometric (LC-MS/MS) analysis of ECM proteins, such as glycosaminoglycans (GAGs), collagen, fibronectin, and laminin in the native trachea and PDTG. **(F)** Micro-CT images of the native trachea, PDTG, and syngeneic tracheal graft (STG) at day 0 and 6 months after tracheal replacement (Tan et al., 2023a).

In 2016, Sun et al. (2016) decellularized tracheae from New Zealand white rabbits using a detergent-enzymatic protocol over seven cycles. 1% aqueous genipin solution was again used as a natural cross-linking agent. The secant modulus (a measure of stiffness) of genipin-treated tracheae was significantly increased (p < 0.05). In an attempt to assess the success of a novel decellularization process, Hung et al. (2016) used a novel Freeze-Dry-Sonication-Sodium dodecyl sulfate (FDSS)-based process on the rabbit trachea involving freeze-drying, sonication, and treatment with SDS. Post-decellularized scaffold transplantation, the rabbits did not survive and died within 7–24 days. The deaths were attributed to the significant collapse of the tracheal tubular structures. Further attempts were made in 2017, Xu et al. (2017) generated a decellularized tracheal scaffold using a laser micropore technique (LMT). The LMT parameters were optimized (17 ms pulse width, 250 lm spot diameter, 10 W output power, and four repeats) for treating the trachea, followed by the detergent-enzymatic method for decellularization. It was observed that the laser micropore technique (LMT) enhanced the porosity of the decellularized trachea matrix, thus making it easier to remove cells from it than untreated trachea. Despite the increased porosity, the LMT-treated trachea maintained its original shape with only mild damage. Butler et al. (2017) developed a new protocol for decellularization that required only 9 days. The tracheas underwent lyophilization before decellularization with 0.25% Triton X-100 and sodium deoxycholate (SDC), followed by RNase (4U/mL) and DNase-I (2kU/mL) for the removal of cellular and nuclear components. All decellularization steps were done in a chamber under vacuum (1 Torr). The decellularized trachea obtained by vacuum-assisted decellularization (VAD) showed decreased DNA content compared to DEM. This technique can accelerate the decellularization process (Figure 2; Butler et al., 2017). The attempt made by Butler et al. (2017) required 9 days whereas in a study conducted by Hong et al. (2018) the total time required for decellularization was only 3 days and 12 h. They used a patented decellularization treatment [US 9,566,369 B2]. It comprised a hypotonic solution, hypertonic solution, protease inhibitors, surfactants, DNase, RNase, peracetic acid and extensive washing in PBS. The average reduction in DNA content was 97.14%. The residual DNA was probably from the retained chondrocytes that were retained in the deep cartilage regions. Tan et al., studied the potential of various techniques for decellularization. The protocols included DEM with continuous agitation, DEM with sonication (DEM-S), DEM with freeze-thaw and lyophilization cycles (DEM-L), time-adjusted DEM (2-h detergent incubation) and a bioreactor-based DEM (DEM-B) (Figure 3; Tan et al., 2023a). Sonication and lyophilization did not show significant advantages, but the bioreactor method showed better preservation of ECM integrity compared to continuous agitation. In 2019, Giraldo-Gomez et al. applied a new technique involving an ultrasonic bath and other chemical agents for decellularization. The tracheas were first incubated in 1% Trypsin-EDTA solution, then in 10% Guanidine solution in Buffer Tris, and then in 4% sodium deoxycholate for 7 h. The tracheas were subjected to three sequential incubations. First, they were immersed in a 1% sodium dodecyl sulfate (SDS) solution for 16 h. Next, they were placed in a 2% tributyl phosphate solution in Buffer Tris for 24 h at room temperature. Finally, they were immersed in 70% ethanol for 24 h. After that, a freeze-thaw process was performed for 72 h. The new protocol removed cellular components and nuclear material, preserving the biomechanical properties of the tracheal tissue after decellularization. Batioglu-Karaaltin et al. (2019) used a combination of physical, chemical, and enzymatic methods for decellularization of rabbit tracheas. All samples were subjected to freeze-drying followed by chemical treatment using deoxycholic acid, Triton X-100, and sodium dodecyl sulfate (SDS). They then treated the samples with deoxyribonuclease (DNase) to remove the nuclear material. The results indicated that lyophilization, combined with deoxycholic acid and deoxyribonuclease treatment, was the most effective method, requiring only 3 days.

In 2021, Sun et al. (2021) designed a vacuum-assisted decellularization (VAD) method to obtain decellularized tracheal segments from male New Zealand white rabbits. Tracheal segments were placed in a vacuum tube system having a pressure of ∼0.96 Mpa. They were treated with a solution comprising 0.25% sodium deoxycholate and Triton X-100, followed by washing in sterile distilled water. These were then treated with 4 U/mL RNAse and 2 kU/mL DNAse. Based on the duration of the decellularization method, it was divided into four groups (native trachea, VAD 8 h, VAD 16 h, and VAD 24 h groups). This method effectively removed cellular components and nuclear material. The VAD-treated tracheal matrix, when seeded with bone marrow-derived endothelial cells (BMECs), demonstrated biocompatibility with promoted neovascularization *in vivo*. Based on these observations, VAD 16 h and VAD 24 h groups showed better results. Moreover, the vascular network formation surrounding the graft facilitated the migration of ciliated epithelial cells onto the tracheal graft. Milian et al. (2021) used different detergents for obtaining decellularized porcine tracheas, including SDS, SDC, and Triton X-100. The experimental groups included 0.25% SDS + 0.2% Triton X-100, 0.25% SDS + 2% Triton X-100, 2% SDS, and 4% SDC. 2% Sodium dodecyl sulfate (SDS) was found to be the most effective detergent, while Other detergents (Triton X-100 and SDC) showed varying degrees of effectiveness but were less optimal compared to SDS. Decellularized tracheas were stored for up to a month using liquid nitrogen to study the impact of cryopreservation on its histological and biomechanical properties. The study suggested that it is a viable method for long-term storage without compromising tissue integrity. Martínez-Hernández et al. (2021) used a combination of antibiotics, chemical and physical agents to achieve the decellularization of rabbit tracheas. The samples were immersed for 5 weeks with continuous stirring in a PBS solution containing 2% sodium dodecyl sulfate, 5% penicillin-streptomycin, and 5% amphotericin B. The decellularized organ displayed minimum chondrocyte remains in the cartilage as well as DNA quantification values < 50 ng in electrophoresis.

**FIGURE 4:**
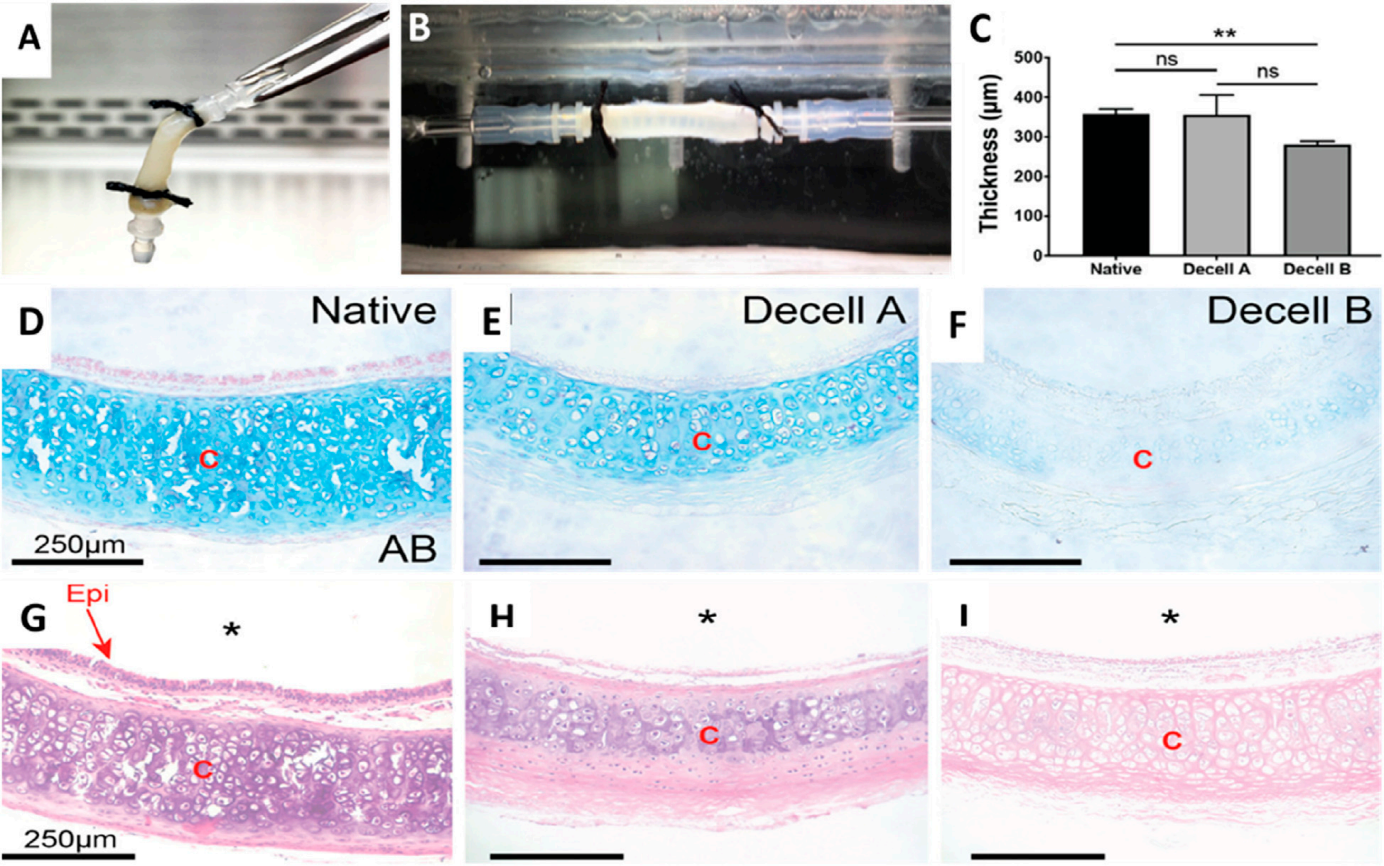
The decellularization process alters the structural and mechanical characteristics of the native rat trachea. **(A)** The gross image of a decellularized rat trachea that has been cannulated. **(B)** Photograph of a decellularized trachea installed in the biaxial mechanical testing apparatus. **(C)** Wall thickness measurements of native, Decell A, and Decell B tracheal segments were conducted using Welch’s t-test. **(D–F)** Alcian blue staining images of native, Decell A, and Decell B rat tracheas depict different glycosaminoglycan levels (scale bar – 250 μm). **(G–I)** H&E images of native, Decell A, and Decell B rat tracheas at ×20 magnification illustrate the varying levels of cellular loss (scale bar - 250 μm) (* implies lumen; “Epi” implies epithelium, and “C” implies cartilage) (Greaney et al., 2023).

Wang et al. (2021) subjected pig tracheal scaffolds to different treatments of decellularization and cryopreservation. One group was decellularized using 3% sodium dodecyl sulfate (SDS). Another was cryopreserved for 3 months. The next group was also cryopreserved for 3 months and then decellularized. The final group was decellularized first, then cryopreserved at −80°C for 3 months. The decellularization process effectively removed epithelial cells and submucosal cells, particularly when applied after cryopreservation. The results suggested that this method preserved the extracellular matrix and cartilage structure better than the reverse order. In 2022, Baranovskii et al. (2022) used a new protocol for the decellularizing human tracheas. The samples underwent five cycles of the freeze and thaw method followed by laser perforation (engraving). The laser operated at 30 W with a velocity of 42.6 cm/s. Circular grooves of 10 μm were engraved 250 μm apart. The laser perforation resulted in regular porosity in the decellularized tracheas without significantly altering the cartilaginous matrix. Moreover, the DNA content of the decellularized tracheal scaffolds averaged 35 ± 12 ng/mg. In 2023, A recent study by Tan et al. highlighted that partially decellularized tracheal grafts (PDTG) promote host-driven regeneration of tracheal neo-tissue. Through microsurgical, transcriptomic, and immunofluorescent analyses, the study compared PDTG neo-tissue with surgical controls and native trachea. Findings revealed that PDTG neo-tissue, containing native airway cell types, maintained microvasculature and neo-epithelium for at least 6 months. Vascular perfusion was established within 2 weeks, facilitating the recruitment of multipotent ABSCs with normal proliferation and differentiation capabilities. As a result, PDTG neo-tissue closely mimics the architecture and functionality of the native trachea while supporting regeneration (Figure 3; Tan et al., 2023a). Wang et al. (2023) compared decellularized tracheal grafts to 3-D printed tracheal grafts. They decellularized the rabbit trachea by incubating it in 4% SDC solution for 4 h followed by treatment with a 1 mol/L sodium chloride solution containing DNase-I (2000 kU/L), for 3 h (23°C). After 7 cycles, the tracheas were washed and kept overnight in a PBS-antibiotics solution (streptomycin, amphotericin B, and penicillin). Polycaprolactone (PCL) was used to create 3D-printed tracheal grafts. The 3D-printed scaffolds had better mechanical properties, while the ones obtained through decellularization offered better biocompatibility and lower immunogenicity. Greaney et al. (2023) isolated tracheas from WT Sprague Dawley rats and decellularized them using two protocols: Decell A (mild) and Decell B (harsh). In protocol ‘Decell A’, they perfused it with the subsequent solutions for decellularization using a peristaltic pump: Antibiotics (2% gentamycin, 10% penn/strep, and 4% amphotericin B) for 5 min; 0.0035% Triton X-100 for 30 min; 1M NaCl for 10 min; PBS for 30 min; 0.1% SDC for 1.5 h; PBS for 30 min; 15 U/mL Benzonase for 1 h; PBS for 30 min; 0.5% Triton X-100 for 10 min. After a final PBS wash, the tracheas were sterilized by perfusion with the antibiotics mentioned above. In Decell B, the tracheas were connected to a peristaltic pump and treated with 25 cycles of 4% sodium deoxycholate for 4 h, and then DNase-I (2000 kU) in 1 M sodium chloride (NaCl) for 3 h after washing in distilled water. While Decell B eliminated almost all of the tissue’s cells, including chondrocytes, Decell A only eliminated stromal and epithelial cells while leaving the cartilage mostly unaltered. Preservation of collagen I and loss of collagen III by ‘Decell A’ made the decellularized tracheas more stiff than native. This suggests that the presence of only stiff collagen I results in reduced circumferential and axial stretch. Tissue mechanics were affected in the axial direction after decellularization (Figure 4; Greaney et al., 2023).

Stocco et al., developed a new protocol using physical (freezing at −20°C for 12 h and lyophilization for 12 h), enzymatic (DNase-I (10,000 kU) and 0.05% trypsin), and chemical (2% Tergitol detergent solution and 0.8% ammonium hydroxide) treatments. 12 cycles of this decellularization process were performed (Stocco et al., 2023). Decellularization successfully removed Alpha-Gal epitopes, which trigger immune responses. But it also resulted in the loss of Glycosaminoglycans (GAGs). Matias et al. used 3 decellularization protocols to obtain acellular scaffolds from canine tracheas. In protocol 1, they immersed the tracheal sections in 4% SDS detergent combined with orbital shaking (60 rpm) (Matias et al., 2023). In protocol 2, the tracheas were immersed in 4% SDS detergent with similar agitation for 8 days followed by incubation in 4% SDS with 12% FBS for 48 h. For protocol 3, the samples were treated with 4% SDS with agitation for 8 days and then immersed in SDS 4% and stored under high pressure in a vacuum (<1,000 Pa) for 120 min. The protocol 3 decellularized samples showed better ECM protein (collagens, GAGs, and PGs) preservation. Moreover, the DNA content of protocol 1 (14.04 ng/mg), protocol 2 (17.58 ng/mg), and protocol 3 (13.5 ng/mg) were lower than 50 ng/mg of tissue. Nahumi et al. (Nahumi et al., 2023) placed the sheep trachea in liquid nitrogen for 5 min, undergoing three cycles of freezing and thawing. The tracheal segments were agitated in a decellularization solution of 0.25% SDC and Triton X-100 for 24 h at 37°C, followed by a PBS wash. The tracheal sections were treated with 150 U/mL DNase I and then left in isopropanol overnight. Three 10-min PBS rinses were then conducted after this. Nearly complete removal of cell nuclei and minimal damage to the ECM were observed. de Wit et al. (2023) developed a new protocol using Supercritical carbon dioxide (37°C, 250 bar) as a potential alternative to DEM. They performed Supercritical carbon dioxide processing as a dynamic run for 12 h. Supercritical carbon dioxide-based decellularization allows for a tuneable process but cannot reduce the number of nuclear remnants below 50 ng/mg of tissue. Guimaraes et al. (2023) decellularized porcine tracheas using a modified DEM treatment. It involved a 10-cycle treatment in 2% sodium deoxycholate detergent 0.02% EDTA, and constant agitation. The amount of DNA dropped to 20 ± 8 ng/mg dry tissue from 850 ± 123 ng/mg, indicating successful decellularization. In 2024, Jiwangga et al. (2024b) applied a detergent-enzymatic-based decellularization technique using 1% sodium dodecyl sulfate (SDS) and 3% hydrogen peroxide (H_2_O_2_) on goat tracheas. Their results showed that the scaffold produced by this method had minimal cell nuclei, preserved ECM, low toxicity, and exhibited high elasticity and tensile strength. The data is summarized in Table 1. The data from these studies support the development of various decellularization methods and techniques to generate effective tracheal scaffolds for addressing prevalent respiratory diseases. The different decellularization technique used in tracheal decellularization is summarized in Table 2.

**TABLE 1:** Various decellularization techniques and agents utilized for tracheal regeneration across different animal models.

Sr No.	Animal Source	Type of Decellularization	Decellularization Agents	Benefits	Drawbacks	Refs
1	Pigs	Chemical, Enzymatic based method	Sodium deoxycholate (SDC), DNase 1	Epithelial cells were completely removed, Scaffold supported the adhesion of epithelial cells and chondrocytes	Chondrocytes werepartially removed	Conconi et al. (2005)
2	Human	Chemical, Enzymatic based method	SDC, DNase 1	99% of the DNA had been removed, ECM histoarchitecture was preserved, bFGF was preserved	Chondrocytes were partially removed, long processing time (30 days) for decellularization	Baiguera et al. (2010)
3	Pigs	Chemical, Enzymatic based method	SDC, DNase 1	The submucosal part was completely decellularized, while apoptotic changes were observed in chondrocytes	Chondrocytes were partially removed, and loss of soluble collagen, and GAGs	Partington et al. (2013)
4	Male Brown Norway rats	Chemical, Enzymatic based method	SDC, DNase 1	Decellularization combined with chemical cross-linking results in a scaffold that retains structural proteins and extracellular matrix components. The basement membrane was preserved	Needs a bioreactor system to fasten the process	Baiguera et al. (2014)
5	New Zealand White rabbits	Physical, Chemical based method	Freeze-drying, sonication, SDS	Soft tissue cells were completely removed	Chondrocytes were partially removed, and mild disruptions of the mucosal layer	Hung et al. (2016)
6	New Zealand White rabbits	Physical, Chemical, and Enzymatic based method	Laser micropore technique, SDS, DNase 1	The decellularized trachea matrix had enhanced porosity, the LMT-treated trachea maintained its original shape	Only mild damage was observed, burning of the trachea could produce cytotoxic compounds	Xu et al. (2017)
7	New Zealand White rabbits	Physical, Chemical, and Enzymatic based method	Freeze-drying, TritonX-100, SDS, DNase 1	Short and effective decellularization method requiring only 3 days, DNA concentration: <50 ng/mg	The cartilage matrix significantly decreased after decellularization, and cellular remnants persisted	Batioglu-Karaaltin et al. (2019)
8	Landrace pigs	Physical, Chemical based method	Ultrasonication, Freeze-thaw, Trypsin-EDTA, Guanidine solution, SDC	The scaffold was completely decellularized, and <0.01 μg/mg DNA was retained	Loss of GAGs and increased stiffness of tissue	Giraldo-Gomez et al. (2019)
9	New Zealand White rabbits	Physical, Chemical based method	Vacuum-assisted technique, Triton X-100, SDC	24 h treated group showed the least cell nuclei, all groups showed similar structural integrity	All groups showed reduced sGAG expression	Sun et al. (2021)
10	Pigs	Chemical based method	SDS, SDC, Triton X-100	2% SDS removed maximum chondrocytes while preserving ECM architecture, and multiple osmotic shocks reduced the harmful effects of SDS	Cryopreservation leads to changes in scaffolds’ properties, long treatment duration	Milian et al. (2021)
11	Human	Physical based method	Freeze-thaw, Laser perforation	Preserved GAG matrix in the remaining regions of the laser-perforated tracheal cartilage	Loss of GAG only in the part immediately surrounding the pores	Baranovskii et al. (2022)
12	Male WT Sprague–Dawley rats	Physical, Chemical, and Enzymatic based method	Decell A method: Perfusion, Triton-X, SDC, Benzonase Decell B method: SDC, DNase	Decell method A removed epithelial and stromal cells while leaving the cartilage intact producing stiffer tissue	Decell B method caused severely compromised ECM structure, including damage to collagen I fibers	Greaney et al. (2023)
13	Sheep	Physical, Chemical, and Enzymatic based method	Freeze-thaw, Triton X-100, SDC, DNase I	Nearly complete removal of cell nuclei with minimal damage to the ECM	Tensile strength was reduced	Nahumi et al. (2023)
14	Landrace pigs	Physical based method	Supercritical carbon dioxide technique, Lyophilization	Method 1 resulted in efficient chondrocyte removal	Method 2 resulted in collagen loss	de Wit et al. (2023)
15	Goat	Chemical based method	SDS, Peroxide (H_2_O_2_)	3% H_2_O_2_ achieved the most decellularization, lowest antigenicity, preserved ECM, low toxicity	3% H_2_O_2_ showed comparatively low-stress resistance	Jiwangga et al. (2024b)

**TABLE 2:** Different decellularization techniques, their components, underlying mechanisms, and roles in tracheal decellularization.

Decellularization type	Components	Mechanism	Benefits	Drawbacks	Refs
Chemical-based decellularization methods	Sodium dodecyl sulfate (SDS)	Ionic and non-ionic detergents (SDS, SDC, and Triton X-100) that solubilize cell membranes by disrupting lipid-lipid interactions	Effective cell removal	Cytotoxic, tends to disrupt native tissue structure, removes GAGs, and damage collagen, difficult to fully remove from tissues	Milian et al. (2021)
	Sodium deoxycholate (SDC)		Cell removal maintains ECM proteins, effectively decellularizes thicker tissues, and	Can damage soft tissues, remove GAGs, can cause agglutination of DNA when used without DNase	Noori et al. (2023)
	Triton X-100		Milder and used for decellularizing softer tissues where preservation of ECM architecture is crucial	Mild, may not completely remove cellular material, limited effectiveness in dense tissues	Tebyanian et al. (2019)
	3-[(3-(cholamidopropyl) dimethylammo- nio]-1- propanesulfonate (CHAPS)	Zwitterionic detergent that solubilizes cell membranes by disrupting lipid-lipid interactions	Has properties of non-ionic and ionic detergents, maintains structural ECM proteins	may not completely remove cellular material, relatively expensive	Petersen et al. (2011)
	Peracetic acid	Disrupts nucleic acids	Used for sterilization	highly oxidative and can degrade ECM proteins	Poornejad et al. (2016)
	Ethylenediaminetetraacetic acid (EDTA)	A chelating agent that binds divalent metallic ions, disrupting cell adhesion to ECM	Breaks cell-matrix adhesions for cell removal	does not directly lyse cells, requires supplementation with other agents, Decreases salt-and acid-soluble ECM proteins	Giraldo-Gomez et al. (2019)
	Hypertonic PBS and hypotonic solutions (Sterile distilled water)	Burst cell membrane by inducing osmotic shock, disrupt DNA-protein interactions	Causes cell lysis, cell removal	Less effective for thick tissues, difficult to standardize concentration of osmotic agent	Milian et al. (2021)
Physical-based decellularization methods	Mechanical agitation	Facilitates chemical exposure and cellular material removal	Causes cell lysis	Cell lysis may initiate prior to exposure to the detergent, resulting in the release of proteases that degrade the ECM	Minnich and Mathisen (2007)
	Freeze-thaw	Intracellular ice crystals disrupt cell membrane	Causes cell lysis, maintains ECM structural properties	Requires supplementation with other methods to remove cellular remnants from scaffold	Neishabouri et al. (2022)
	Perfusion	Uses native vasculature of the organ to deliver decellularization agents	Effectively decellularizes large organs	Can damage thin vessels if pressure is high	Greaney et al. (2023)
	Lyophilization	Removes water from tissues by freezing and then subjecting them to low-pressure sublimation	Cell removal	Can make tissues brittle	Sun et al. (2016)
	Vacuum assistance	Uses negative pressure to enhance the infiltration of decellularization agents	Effectively decellularizes dense organs, accelerates and improves detergent treatment	Requires supplementation with chemical methods to damage cells	Sun et al. (2021)
	Laser micropore technique	Uses a laser to make pores on the surface to enhance the infiltration of decellularization agents	Effectively decellularizes dense organs, maintains mechanical properties	Damages heat-sensitive ECM proteins and glycosaminoglycans, requires advanced laser equipment	Xu et al. (2017)
	Supercritical carbon dioxide	CO_2_ is applied at pressures above 7.40 mpa and temperatures above 31.1^∘^C	Maintains ECM proteins and mechanical properties, pressure applied can burst cells, no need for additional washing steps	Pressure can damage ECM	de Wit et al. (2023)
Enzymatic-based decellularization methods	Endonuclease (Benzonase, DNase and RNase)	Hydrolysis of the interior bonds in ribonucleotide and deoxyribonucleotide chains	Degradation of RNA or DNA	Relatively expensive, time-consuming for dense tissues	Partington et al. (2013)
	Phospholipase	Damages the phospholipid components	Preserves the collagen	May damage lipid components of the ECM	Neishabouri et al. (2022)

## 5 Retention of native tracheal ECM integrity post-decellularization

The ECM of the native trachea is preserved using optimized chemical, enzymatic, and physical treatments, including mild detergents, nucleases, agitation, and perfusion, with careful control of parameters like detergent concentration, enzymatic activity, and exposure time. These methods avoid harsh chemicals, cryo-processing, and freeze-thaw cycles that could damage key structural proteins such as collagen, elastin, and glycosaminoglycans, which are vital for maintaining the ECM’s functionality and bioactivity (Jiwangga et al., 2024a). On the other hand, major histocompatibility complex (MHC) class I and II molecules, which are cell-derived and capable of triggering immune responses, are effectively removed during the decellularization process. This is achieved through similar treatments that solubilize cellular membranes and DNA, ensuring that the ECM remains immunologically inert while preserving its structural integrity and biochemical properties essential for tissue engineering and regenerative medicine applications (Bogan et al., 2016; Wang et al., 2023).

Any technique that disrupts and removes the cellular component of tissues will, to some extent, alter the ultrastructure and composition of the tracheal ECM. Lower pH conditions during decellularization have been found to better preserve ECM components like elastin, fibronectin, and laminin, which are crucial for maintaining tissue architecture and reducing host response upon implantation (Tsuchiya et al., 2014). Moreover, the extent of ECM preservation also depends on the chemicals used in decellularization. Zang et al. (2012) observed a 47% loss of GAGs in the decellularized trachea after treatment with 5 cycles of 4% SDC. Matias et al. (2023) compared a 4% SDS-based decellularization protocol with the same under vacuum conditions. Results showed higher levels of collagens, sulfated GAGs, and proteoglycans in the sample decellularized under vacuum conditions. Although lower than the native tissue, the use of vacuum ensured superior preservation of ECM proteins. Understanding the effect of decellularization on the ECM is essential, and can be studied using techniques such as SDS-PAGE, Western blot, colorimetric assays, histology, and mass spectrometry. Morphological characterization can be achieved using SEM, AFM, and TEM imaging (Vilaça-Faria et al., 2024). Compromised ECM micro-architecture and loss of ECM proteins are some factors that can impact the end product’s viability as a tracheal substitute. The weakened ECM scaffold can collapse after transplant in the recipient if there is a considerable loss of collagen. Collagen is responsible for the flexibility of the trachea, and changes in collagen content can lead to stiffness. GAGs are another important component that helps prevent inflammation (Vilaça-Faria et al., 2024). This property is essential for organ transplants. Recellularization is also affected as the stem cells introduced into the ECM exhibit morphological changes that align with the lineage of the tissue from which the ECM is derived, independent of the origin of the seeded cells (Moffat et al., 2022). The basement membrane complex (BMC) is an essential element of the ECM as it facilitates cellular growth. Most detergents change the ultrastructure and composition of the BMC, and this can affect the behaviour of seeded endothelial cells (Faulk et al., 2014).

## 6 Recellularization

Recellularization is the most crucial and complex step after the decellularization process. Cells are the essential pillar in tissue engineering for achieving the regeneration of functional tissues. Recellularization is the process of repopulating acellular extracellular matrix (ECM) scaffolds of tissues or organs with tissue-specific cells or stem cells (such as embryonic stem cells (ESCs) or induced pluripotent stem cells (iPSCs)). The goal is to rebuild the organ’s micro-anatomy and restore its specific function (Hillebrandt et al., 2019). Cell seeding is an important step in recellularization. It aims to position an appropriate cell mixture in the scaffold to simulate the natural distribution of cells in organs and tissues. Ideally, the cells must be survivable, functional, easy to harvest, non-immunogenic, and similar to the cells in the native tissue. In the trachea, the mechanical functions are carried out by both the ECM as well as the contractile cells of the trachealis muscles.

In the native trachea, chondrocytes occupy the external surface, while a ciliated pseudostratified columnar epithelium lines the inner surface. Thus, chondrocytes and epithelial cells constitute the main cell types. Stem cells are commonly used to generate various cell types in the scaffold. Amniotic fluid stem cells, ESCs, Mesenchymal stem cells (MSCs), iPSCs, human umbilical cord blood-derived MSCs, and bone marrow-derived MSCs can be used to recellularize the tracheal scaffold. While some cells have the potential to initiate an immune response post-transplantation, MSCs lack MHC-II and have reduced expression levels of MHC-I proteins (Romieu-Mourez et al., 2007). Haykal et al. (2013) conducted a study that showed that MHC proteins may persist in the cavities of submucosal glands even after decellularization. This could potentially activate CD4^+^ T cells and lead to proliferation. Mesenchymal stem cells and tracheal epithelial cells were used to regenerate the decellularized tracheal scaffolds. They observed that this approach produced a suppressive outcome on CD4^+^ T cells and led to an upsurge in CD4^+^CD25+Foxp3+ regulatory T cells, indicating an active immune-modulation mechanism by recellularization. Adipose tissue-derived MSCs (AT-MSCs) and bone marrow-derived MSCs (BM-MSCs) are commonly used sources for mesenchymal stem cell production in tracheal tissue engineering (Batioglu-Karaaltin et al., 2019). Giraldo-Gomez et al., seeded human adipose-derived mesenchymal stem cells (hADMSCs) for the recellularization of the scaffold. They obtained subcutaneous adipose tissue from patients and successfully cultured the hADMSCs from it (Giraldo-Gomez et al., 2019).

Epithelial cell seeding before orthotopic transplantation is necessary to avoid bacterial/fungal contamination. iPSCs are an important autologous cell source that is well-accepted ethically. These iPSC-derived airway epithelial cells can be used to promote re-epithelialization. Airway stem/progenitor epithelial cells (AECs) secrete factors that increase the tracheal epithelial cell repair and regeneration and initiate the epithelial-to-mesenchymal transition (EMT) process. Moreover, Interleukin (IL)-10, produced by AECs, suppresses pro-inflammatory cytokines including tumor necrosis factor TNF-α and IL-1β (Kardia et al., 2017). Besides stem cells, bone marrow cells (BMCs) cultured in endothelial differentiation medium can also differentiate to form endothelial cells. Butler et al. (2017) used primary human bronchial epithelial cells (HBECs) obtained from donor human trachea to seed the decellularized human tracheal scaffolds. Migration of the recipient’s tracheal epithelial cells is possible post-transplantation but would take too much time. Seeding of epithelial cells in the tracheal scaffold before transplantation is crucial, especially for long grafts. Endothelial cells from the thoracic aorta lumen, normal human bronchial epithelial (NHBE) cells, and HM1-SV40 cells can also be used (Sun et al., 2021).

Chondrocytes can be directly isolated from the organism’s cartilage (human, pig, rabbit, mouse, rat, etc.) or differentiated from MSCs. Primary chondrocytes in preclinical studies are usually harvested from tracheal, nasal, coastal, and auricular cartilage. Xu et al. (2017) harvested auricular cartilage from New Zealand white rabbits to isolate chondrocytes, while Baranovskii et al. (2022) used nasal chondrocytes. Growth factors can be used to stimulate MSC differentiation into chondrocytes. Insulin-like growth factor (IGF) increases GAG production, while Fibroblast growth factor (FGF) uses the Wingless (Wnt) signal to induce chondrogenesis differentiation (Le et al., 2020). The transforming growth factor TGF-β also stimulates chondrogenesis of MSCs. TGF-β3 in particular, activates chondrogenesis differentiation of BM-MSCs. BM-MSC was harvested from rabbit femur. These were differentiated into chondrocytes in the presence of 1% sodium pyruvate, dexamethasone, human transforming growth factor-β1, ascorbic acid, and 10% insulin-transferrin-selenium (ITS-premix) (Bae et al., 2018). Chondrogenic gene expression levels were checked for ACAN, Col1α1, Col2α1, and SOX9 to confirm the differentiation of the bMSCs. The analysis showed that all of the chondrogenic gene expressions were much higher in the differentiated MSCs compared to the ones that were not differentiated. The residual MSCs assisted in the rapid angiogenesis. However, mesenchymal stem cell-derived chondrocytes are a better option for cell seeding. BMP2 and BMP7 are bone morphogenetic proteins (BMPs) that also activate chondrogenic differentiation of MSCs (Scarfì, 2016). Even though there are different cartilage types (hyaline, fibrocartilage, and elastic), the kind of chondrocytes used for recellularization does not have any significant impact.

Before cell seeding, the scaffolds are sterilized and cultured in media containing DMEM-F12, FBS, antibiotics solution, etc., to facilitate biocompatibility. Moreover, the scaffolds can be pre-treated with growth factors or cytokines that promote cell adhesion, proliferation, and differentiation. Multiple recellularization techniques can be used to seed different cell types for optimal results. Recellularization can be done through the injection of primary pulmonary and endothelial cells (Petersen et al., 2010). Cells such as chondrocytes can be injected into the tracheal rings. Additionally, epithelial cells are seeded onto the mucosal surface of the acellular scaffolds. Static seeding involves manually pipetting the cells onto the scaffold’s surface (Milian et al., 2021). Another approach involves culturing cells as a single layer or sheet. Once the layer is fully formed, it is harvested and wrapped around the scaffold. Recent studies indicate that using laser perforation increases the porosity of the cartilage surface, allowing efficient cell colonization. The cell-LDTM constructs exhibited a relatively homogeneous cell distribution across both the micropores and bilateral surfaces (Baranovskii et al., 2022). Hydrogel encapsulation helps retain cells in place and provides a conducive environment for growth. Cell attachment on all scaffold surfaces can be effectively achieved through fibrin encapsulation (de Wit et al., 2023). Perfusion recellularization involves perfusing the scaffold with a cell suspension. This can be achieved via the vascular network or another hollow structure such as the bronchial system. In the lungs, the tracheobronchial tree is commonly used to supply the mesenchymal stem cells.

To achieve better recellularization, bioreactors can also be used as an environment that facilitates the recellularization and maturation of organs such as trachea. The body serves as the ideal bioreactor, providing the necessary *in vivo* conditions for an engineered neo-organ to develop, but an *in-vitro* bioreactor is required to create similar conditions. The tracheal scaffold suspended in the bioreactor is attached to a ventilator that corresponds to the animal’s volume and respiratory rate to mimic the mechanical properties during recellularization (Crabbé et al., 2015). Thus, bioreactors can accurately monitor and regulate environmental factors, unlike static culture conditions.

**FIGURE 5:**
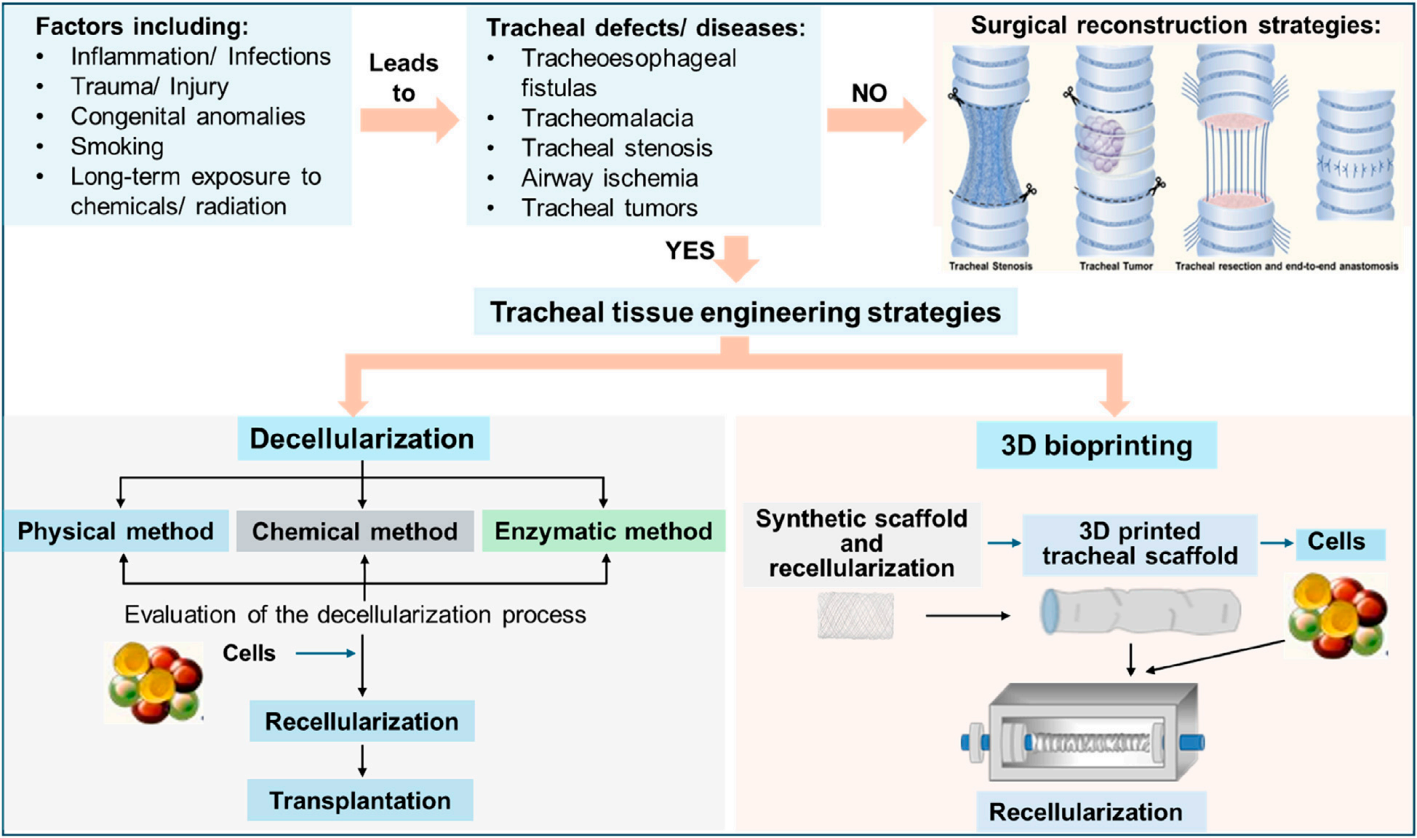
Schematic showing the overview of tracheal defects and tracheal tissue engineering approaches.

Various techniques such as MTT, water-soluble tetrazolium salt (WST-1), flow cytometry, immunohistochemistry (IHC), H&E (hematoxylin and eosin), Alcian blue, real-time-polymerase chain reaction (RT-PCR), and scanning electron microscopy (SEM) have been commonly used for evaluating cell viability after seeding. The MTT and WST-1 assays rely on cellular mitochondrial dehydrogenases to cleave the tetrazolium salts and form formazan. Flow cytometry confirms the identity of the cells used in the repopulation of the scaffold. Immunohistochemistry-based techniques are also used to evaluate recellularization. H&E staining is used to assess the biocompatibility of the acellular tracheal scaffold and Alcian blue staining is used to check the differentiation of chondrocytes. Real-time PCR has also been used to confirm the differentiation of MSCs into tracheal cells (Nahumi et al., 2023). SEM can be used for imaging of the cells and to check their adherence to the scaffold, following evaluation of the recellularization, then, can be transplanted into the host. The schematic of tracheal defects/diseases, and tracheal tissue engineering strategies are shown in Figure 5.

## 7 Post transplantation

For successful tracheal tissue transplantation, the grafts must have adequate mechanical properties, blood supply, lining with ciliated epithelium, and no immunogenicity (Bae et al., 2018). Re-epithelialization ensures the integrity of the scaffold is maintained and prevents infection, formation of granulation tissue, and stenosis post-transplantation. Additionally, the chondrocytes secrete glycosaminoglycans, collagen, and elastin, which improve mechanical strength. Go et al. (2010) transplanted decellularized tracheal graft in pigs. Their study revealed that the group containing BM-MSC-derived chondrocytes and epithelial cells did not show any signs of tracheal ischemia or collapse.

Mechanical properties can be maintained with the help of additional components. This prevents necrosis, stenosis, and collapse of the transplant. Sun et al. confirmed that the decellularized rabbit tracheal graft caused no significant foreign body reaction or rejection after implantation in rats. Moreover, the genipin-treated matrices generated strong angiogenic responses *in vivo* without collapsing (Sun et al., 2016). Decellularized rabbit tracheal scaffold transplantations performed in New Zealand White rabbits resulted in the death of all the rabbits within 7–24 days of transplantation due to suffocation. Necrosis and significant collapse in the tracheal tubular structure were observed, but there were no signs of rejection (Hung et al., 2016). The *in vivo* findings from the transplanted laser micropore technique (LMT) treated scaffolds indicated that the structures were capable of developing fully formed tubular cartilage, along with improved mechanical strength, in comparison to the native trachea (Xu et al., 2017).

Besides mechanical stability, an appropriate blood supply is also needed to support the scaffolds *in vivo*. This ensures that the cells are provided with sufficient nutrient and oxygen supply. Lack of functional vasculature can result in hypoxia and insufficient nutrient supply which will halt further growth of cells and blood vessels (Yang et al., 2020). Vascularization is also critical for the success of tracheal scaffold post-transplantation. It occurs soon after transplantation and involves two processes: angiogenesis and vasculogenesis. In angiogenesis, blood vessels arise from already existing vasculature. In vasculogenesis, blood vessel formation takes place when precursor cells mature and differentiate to give rise to endothelial cells. Due to its complex vascular arrangement, direct revascularization is unsuitable for tracheal scaffolds. Revascularization can be achieved by heterotrophic implantation of the graft. The allograft was implanted in the recipient’s left forearm and later transferred to the orthotopic position. They reported that the tracheal cartilage was encased by recipient vasculature and a lining of mucosal epithelium originating from the recipient. The cartilaginous trachea’s viability was preserved however, the posterior membranous part of the trachea showed avascular necrosis (Delaere et al., 2010). Another technique for revascularization is to wrap the graft with viable tissue, as this stimulates microvesicular ingrowth. Klepetko et al. (2004) used the greater omentum and transferred the donor trachea into the recipient’s abdomen. This technique showed good vascularisation of the tracheal wall. A radial forearm fascial flap can also be used to initiate revascularization (Delaere et al., 1999).

Reduction of allo-antigenicity of allotransplants is necessary to avoid immune rejection after transplantation. The physical process of transplantation can induce stress and injury response, which alters the gene and protein expression levels within donor tissue. This leads to an immunological response from the recipient. Cells of the host’s immune system can recognize many potential damage-associated molecular patterns (DAMPS), such as heat shock proteins, reactive oxygen species, heparin sulfate, etc. Which then triggers the activation of the inflammasome that initiates inflammatory responses through the production of proinflammatory and chemoattractant cytokines, tumor necrosis factor (TNF), interleukins (IL-1 and IL-6), and type I interferons (Wood and Goto, 2012). These proinflammatory cytokines induce human leukocyte antigens (HLA) class II expression in the pulmonary endothelial cells (Bery and Hachem, 2020). Another factor that leads to immune rejection is allorecognition by T-cells. Antibody-mediated rejection often occurs a few days post-transplantation. The host’s T-cells respond to mismatched major histocompatibility complex (MHC) molecules of the donor graft and recipient’s body. Antibody-mediated rejection occurs when the host’s T-cells respond to mismatched human leukocyte antigens (HLA) class II on donor antigen-presenting cells. This increases pro-inflammatory cytokines secretion of IL-6, IL-11, and IL-8 (Kenta and Takaaki, 2020). Additionally, the formation and activation of membrane attack complexes (MACs) damages endothelial cells by exposing the basement membrane and activating the coagulation cascade. This can result in thrombosis and infarction (Bery and Hachem, 2020). Antibody-mediated rejection of the scaffolds can cause negative effects such as hemorrhage and necrosis. Thus, graft accommodation needs to be established. This state of accommodation occurs when a transplanted organ can withstand immune-mediated damage and maintain functionality, even when anti-donor antibodies are present. This may require several weeks of anti-A/B antibody exposure. Highly sensitive and specific HLA antibody detection assays have been developed and used. Eliminating these T-cells is a commonly used approach. Intensive immunosuppression targeting T-cell proliferation and activity is administrated to the patient. Treatment options include corticosteroids, plasmapheresis, intravenous Immunoglobulin (IV-IG), Rituximab, proteasome, and complement inhibition (Bery and Hachem, 2020). The success of the scaffold after transplantation depends on how effectively the decellularization and recellularization process is carried out. The selection method can significantly impact how the body responds to biological support structures. The treatments should ensure the removal of proteins that might lead to immune rejection. The cell types should be chosen based on their angiogenic and non-immunogenic properties. Therefore, several factors need to be considered to achieve successful post-transplantation of tissue or organ grafts.

## 8 Challenges and future scope

Decellularization is one of the best approaches for producing scaffolds, but it has some drawbacks: The unstable mechanical properties of the scaffold are one of them. It is of utmost importance to preserve the mechanical attributes of the trachea in the decellularized scaffold. In particular, a lack of stiffness in the tracheal scaffold may lead to failure of the tracheal graft due to deformation and obstructed airway. This can lead to death caused by suffocation (Hung et al., 2016). Both chemical and mechanical methods can negatively alter the structural proteins of the ECM. Most protocols call for the use of SDS. However, this detergent has been shown to damage the ECM’s microstructure by altering the collagen arrangement. So, more standardized protocols are required to ensure that the SDS does not alter the structure of the cartilage region.

The ideal decellularization process should completely remove cells from the extracellular matrix (ECM) while preserving critical matrix components. However, achieving 100% preservation of ECM components has not yet been possible. Although complete decellularization was observed in the epithelial region, chondrocytes remained in the deeper spaces using almost all techniques. The thickness of the tracheal cartilage makes it challenging for the chondrocytes in the lacunae to be exposed to the chemical agents (Partington et al., 2013). A balance is required between the detergent exposure time and the level of decellularization. Stronger detergents can be used and the exposure time can be extended, but this may cause cell toxicity in the scaffold, decreasing the recellularization capacity (Rieder et al., 2004). Assays need to be developed for measuring the residual chemicals in the decellularized scaffold material. If the sterilization steps are not carried out properly, the chemicals and detergents may persist in the scaffold. It is necessary to find optimal sterilization protocols for the removal of residual detergent from the decellularized tissue. Ionizing radiation such as low doses of gamma irradiation (<15 kGy), ethylene oxide exposure, and supercritical carbon dioxide exposure are some techniques currently used to sterilize scaffolds (de Wit et al., 2023). Further novel analyzing techniques should be developed for the tissues based on their future point of function. Most researchers use Young’s modulus to define the stiffness of the scaffold. However, Greaney et al. (2023) suggested that using non-linear constitutive equations is more suitable to explain the inherently nonlinear mechanical behavior of the cartilage ring, axial connective tissue, and the trachealis muscles. Overcoming the long processing time is another challenge as most decellularization protocols take place over 4–5 weeks. Decellularization is usually a long and intricate process, with multiple steps, using complex combinations of chemical, physical and enzymatic agents. However, a reduction in processing time can be achieved via automation. Multiple scaffolds cannot be produced simultaneously, and the methods have low feasibility due to the large amounts of waste produced. More research is required to improve the scalability and feasibility of the process. This could be achieved through fewer wash steps, reduced number of treatment cycles, and reusing reagents.

In addition to the challenges of decellularization, the process of recellularization also presents significant obstacles to repopulating the tissue. These challenges include the intricate nature of the scaffold, the difficulties in inserting cells into various sections of the scaffold, the need for diverse types of cells, and the uncertainty regarding the functionality and viability of the cells after organ transplantation (Neishabouri et al., 2022). The graft needs to facilitate the development of chondrocytes, epithelial cells, and endothelial cells to create cartilage tissue, epithelium, and blood vessels, respectively. Cell seeding of the entire organ is difficult because of its large surface area. Seeding cells in the deeper layers of the tracheal scaffold such as cartilage proves very tricky. For the survival and growth of the recellularized scaffold *in vivo*, a proper blood supply is required. However, achieving adequate vascularisation can be very difficult. More focus is required on vascular network reconstruction since restoring blood circulation after implantation is necessary. The arrangement of blood vessels in the trachea is very complex. Thus, revascularization of these tissues is a challenge. The addition of angiogenic and growth factors, especially the vascular endothelial growth factor (VEGF) and revascularization of the matrix can help induce adequate vascularization (Neishabouri et al., 2022). Recellularization and culturing within decellularized scaffolds need to be further explored and optimized. Using specific cells such as MSCs, vascular endothelial cells, and endothelial colony-forming cells can increase the angiogenicity of the tissue-engineered organ as they possess inherent angiogenic behaviors (Yang et al., 2020). A proper environment is needed to self-assemble capillary networks that provide the necessary environmental cues. This can be provided through *in vivo* vascularization as the tissues in the body can successfully invade and vascularize a pro-angiogenic implant. Vascularised 3-D cell steroids can be developed. 3D-fabrication approaches can be designed to print the pre-vasculature onto the scaffold directly. Immune rejection can hinder the success of the scaffolds’ functional implantation into the host. Hyperacute rejection usually occurs when patients show the presence of preformed alloantibodies during transplantation. Gal epitope mismatched human leukocyte antigens, presence of foreign chemicals, and damage-associated molecular patterns (DAMPS) of the allotransplants are some factors that contribute to immune rejection. Stahl et al. (2018) used α-Gal knockout pig organs for transplantation. Results showed that the decellularized scaffold from the genetically modified pigs exhibited a reduced immune response compared to normal pig lungs, suggesting that the α-Gal modification effectively lowers the risk of immune rejection. However, further refinements are necessary to optimize these scaffolds for clinical use in transplantation.

**FIGURE 6:**
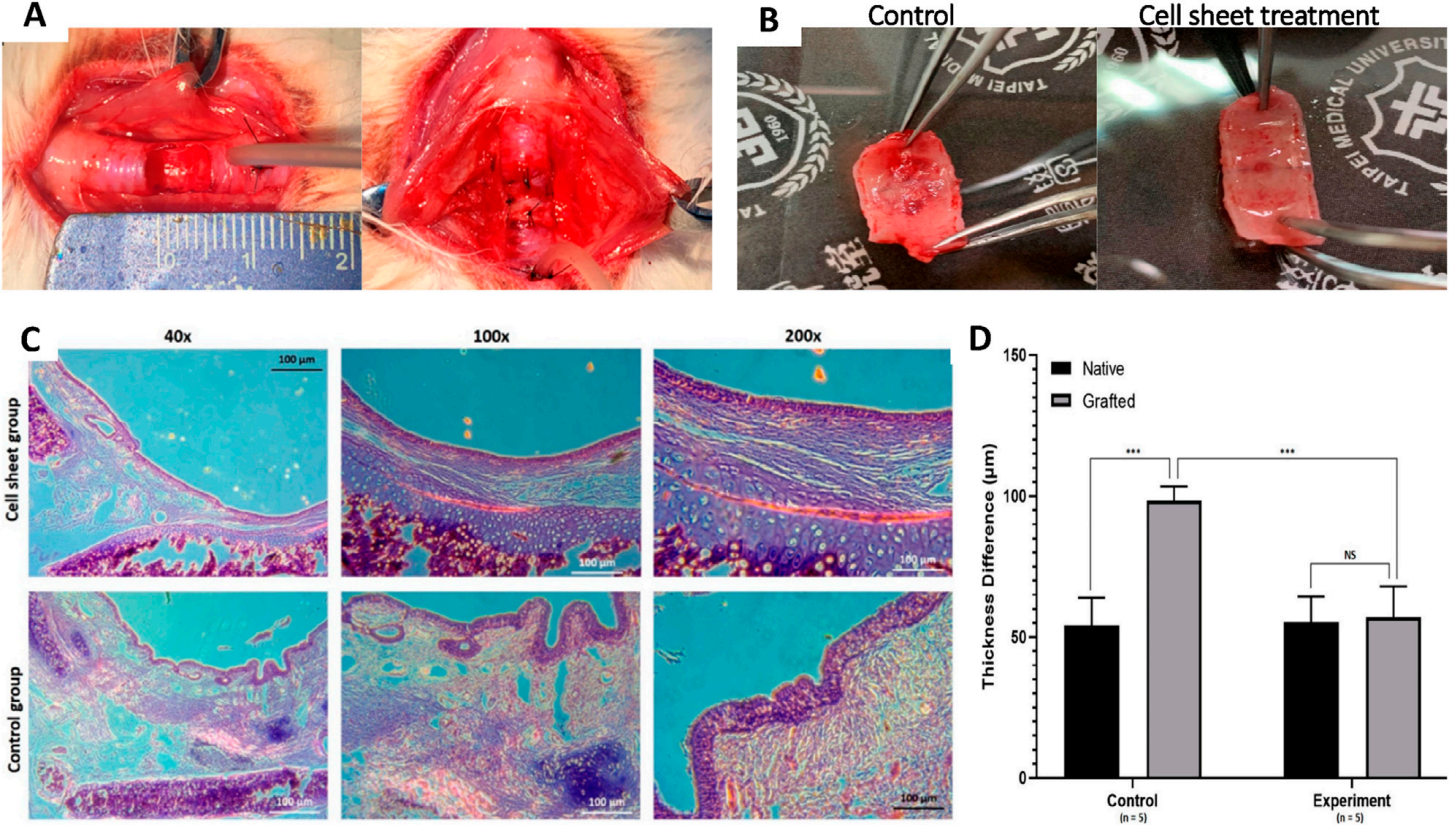
A novel approach combining partial decellularization and cell sheet engineering for tracheal tissue regeneration in a rabbit model. **(A)** A semicylindrical lesion was made on the anterior part of the trachea, followed by autologous transplantation of a partially decellularized tracheal graft integrated with a nasal epithelial cell sheet for reconstructing the trachea. **(B)** A gross evaluation of the luminal aspect of the transplanted graft between the control group and the experimental group treated with the cell sheet. **(C)** H&E staining of transplanted grafts in both control and cell sheet groups at different magnifications. **(D)** A graphical comparison demonstrating significant differences in mucosal layer thickness between the control and experimental groups (Dang et al., 2021).

Thus, despite encouraging results from the decellularized tracheal scaffolds, multiple challenges must be overcome to produce a fully function scaffold that can be clinically applicable. Moreover, to improve the decellularization process, various sensors can be used to collect data at each step in the process. This will help in monitoring the scaffold’s progress during the treatment process and adjust the parameters accordingly. Recent progress in the field of tissue engineering has introduced the concept of partial decellularization. Dang et al., showed that partial decellularization-based tracheal scaffold seeded with autologous nasal epithelial cell-derived sheet. This allows for the development of tracheal scaffolds supporting the formation of autologously-derived tracheal epithelial cells while maintaining mechanical properties (Figure 6; Dang et al., 2021). Furthermore, while allograft implantation leads to CD8^+^ T-cell infiltration, which is associated with rejection, this is not observed with the partially decellularized tracheal grafts. Consequently, we can infer that partial decellularization removes allograft immunogenicity (Tan et al., 2023b).
